# Supplementary material for: Molecular Tumor Board-Guided Targeted Treatments for Biliary Tract Cancers in a Publicly Funded Healthcare System
Source: Curr Oncol. 2025 Jan 31;32(2):80. doi: 10.3390/curroncol32020080 (PMC11854319; doi:10.3390/curroncol32020080)

Supplementary Materials to Molecular Tumor Board-Guided Targeted Treatments for Biliary Tract Cancers in a Publicly Funded Healthcare System

Table S1

Molecular Tumor Board documentation.

**Category**	**Consideration**
Drivers	Mutations, copy number variations, structural variations including fusions
Genome-wide biomarkers	Tumor Mutational Burden (TMB), Mismatch Repair Deficiency (MMRD), Homologous Recombination Deficiency (HRD)
Targets	Alterations indicating drug resistance or sensitivity, as well as potentially available clinical trials
Conclusions	Treatment recommendations based on targetable alterations and patient history

Table S2

Recommendations for targeted treatments based on OncoKB.

ID	Target	Variant type	Possible therapies
BTC0010	*FGFR2*	Fusion	Futibatinib; Pemigatinib; RLY-4008
BTC0011	*MET*	Amplification	Capmatinib; Crizotinib; Tepotinib; Telisotuzumab Vedotin
BTC0013	MSI	MSI-H	Pembrolizumab; Ipilimumab + Nivolumab; Nivolumab; Dostarlimab + Carboplatin + Paclitaxel
BTC0013	*PIK3CA*	H1047R	Alpelisib + Fulvestrant; Capivasertib + Fulvestrant
BTC0013	TMB	TMB-H	Pembrolizumab
BTC0014	*MDM2*	Amplification	Brigimadlin; Milademetan
BTC0014	TMB	TMB-H	Pembrolizumab
BTC0015	*BRAF*	V600E	Vemurafenib; Vemurafenib + Atezolizumab + Cobimetinib; Dabrafenib + Trametinib; Encorafenib + Cetuximab; Dabrafenib; Encorafenib + Binimetinib; Trametinib; Vemurafenib + Cobimetinib; Vemurafenib, Dabrafenib; Encorafenib + Panitumumab; Selumetinib
BTC0016	*PTEN*	Deletion	Capivasertib + Fulvestrant
BTC0018	*FGFR2*	Fusion	Futibatinib; Pemigatinib; RLY-4008
BTC0018	*IDH1*	R132H	Ivosidenib; Olutasidenib; Vorasidenib
BTC0019	*KRAS*	G12D	Cobimetinib, Trametinib; RMC-6236
BTC0020	*EGFR*	Amplification	Cetuximab, Cetuximab + Chemotherapy, Panitumumab, Panitumumab + Chemotherapy
BTC0024	*ERBB2*	Amplification	Ado-Trastuzumab Emtansine; Lapatinib + Capecitabine, Lapatinib + Letrozole; Margetuximab + Chemotherapy; Neratinib, Neratinib + Capecitabine; Trastuzumab + Pertuzumab + Chemotherapy; Trastuzumab + Tucatinib + Capecitabine; Trastuzumab

			Deruxtecan; Trastuzumab, Trastuzumab + Chemotherapy; Tucatinib + Trastuzumab; Pembrolizumab + Trastuzumab + Chemotherapy; Trastuzumab + Chemotherapy; Trastuzumab + Pertuzumab; Lapatinib + Trastuzumab; Trastuzumab + Carboplatin-Taxol
BTC0024	*MET*	Amplification	Capmatinib; Crizotinib; Tepotinib; Telisotuzumab Vedotin
BTC0025	TMB	TMB-H	Pembrolizumab
BTC0026	*FGFR2*	fusion	Futibatinib; Pemigatinib; RLY-4008
BTC0027	*PIK3CA*	E545K	Alpelisib + Fulvestrant; Capivasertib + Fulvestrant
BTC0028	*IDH1*	R132C	Ivosidenib; Olutasidenib; Vorasidenib
BTC0033	*ERBB2*	Amplification	Ado-Trastuzumab Emtansine; Lapatinib + Capecitabine, Lapatinib + Letrozole; Margetuximab + Chemotherapy; Neratinib, Neratinib + Capecitabine; Trastuzumab + Pertuzumab + Chemotherapy; Trastuzumab + Tucatinib + Capecitabine; Trastuzumab Deruxtecan; Trastuzumab, Trastuzumab + Chemotherapy; Tucatinib + Trastuzumab; Pembrolizumab + Trastuzumab + Chemotherapy; Trastuzumab + Chemotherapy; Trastuzumab + Pertuzumab; Lapatinib + Trastuzumab; Trastuzumab + Carboplatin-Taxol
BTC0034	*MDM2*	Amplification	Brigimadlin; Milademetan
BTC0038	*ERBB2*	FISH	Ado-Trastuzumab Emtansine; Lapatinib + Capecitabine, Lapatinib + Letrozole; Margetuximab + Chemotherapy; Neratinib, Neratinib + Capecitabine; Trastuzumab + Pertuzumab + Chemotherapy; Trastuzumab + Tucatinib + Capecitabine; Trastuzumab Deruxtecan; Trastuzumab, Trastuzumab + Chemotherapy; Tucatinib + Trastuzumab; Pembrolizumab + Trastuzumab +

			Chemotherapy; Trastuzumab + Chemotherapy; Trastuzumab + Pertuzumab; Lapatinib + Trastuzumab; Trastuzumab + Carboplatin-Taxol
BTC0039	*MDM2*	Amplification	Brigimadlin; Milademetan
BTC0042	*IDH1*	R132C	Ivosidenib; Olutasidenib; Vorasidenib
BTC0045	MSI	MSI-H	Pembrolizumab; Ipilimumab + Nivolumab; Nivolumab; Dostarlimab + Carboplatin + Paclitaxel
BTC0045	TMB	TMB-H	Pembrolizumab
BTC0052	*PTEN*	Deletion	Capivasertib + Fulvestrant
BTC0054	*IDH1*	R132C	Ivosidenib; Olutasidenib; Vorasidenib
BTC0057	*MDM2*	Amplification	Brigimadlin; Milademetan
BTC0064	*IDH1*	R132H	Ivosidenib; Olutasidenib; Vorasidenib
BTC0064	*PIK3CA*	E542K	Alpelisib + Fulvestrant; Capivasertib + Fulvestrant
BTC0070	*FGFR2*	Fusion	Futibatinib; Pemigatinib; RLY-4008
BTC0077	*KRAS*	G12R	Cobimetinib, Trametinib; RMC-6236
BTC0078	*MDM2*	Amplification	Brigimadlin; Milademetan

Table S3

Recommendations for targeted treatments from the molecular tumor board for alterations not in OncoKB.

**ID**	**Gene**	**Variant_type**	**Therapy**	**OncoKb**
BTC0011	*CDK6*	Strong amplification	CDK4/6 inhibitor	New biomarker
BTC0017	*PBRM1*	Indel	PARPi	New biomarker

BTC0024	*CDK6*	Strong amplification	CDK4/6 inhibitor	New biomarker
BTC0014	*STK11*	Indel	mTOR inhibitor	New biomarker
BTC0014	*STK11*	Indel	DDR repair	New biomarker
BTC0034	*MTAP*	Deletion	PRMT5 inhibitor	New biomarker
BTC0037	*HAS2*	Strong amplification	HAS2 inhibitor	New biomarker
BTC0036	*MTAP*	Deletion	PRMT5 inhibitor	New biomarker
BTC0038	*MTAP*	Deletion	PRMT5 inhibitor	New biomarker
BTC0044	*MTAP*	Strong amplification	PRMT5 inhibitor	New biomarker
BTC0042	*MTAP*	Strong amplification	PRMT5 inhibitor	New biomarker
BTC0056	HRD	HRD	PARPi	New biomarker
BTC0059	*MTAP*	CNV	PRMT5 inhibitor	New biomarker
BTC0058	*MTAP*	CNV	PRMT5 inhibitor	New biomarker
BTC0061	*HRD*	HRD	PARPi	New biomarker
BTC0067	*MTAP*	Deletion	PRMT5 inhibitor	New biomarker
BTC0062	*RASGRF2*	Fusion	MEK inhibitor	New biomarker
BTC0063	*MTAP*	Deletion	PRMT5 inhibitor	New biomarker
BTC0049	*BAP1*	SNV	PARPi	New biomarker
BTC0069	*MTAP*	Deletion	PRMT5 inhibitor	New biomarker
BTC0069	*MTAP*	Deletion	MAT2A inhibitor	New biomarker
BTC0072	HRD	HRD	PARPi	New biomarker
BTC0078	*CCNE1*	Strong amplification	CCNE1 targeting trial	New biomarker
BTC0080	HRD	HRD	PARPi	New biomarker
BTC0077	*MTAP*	Deletion	PRMT5 inhibitor	New biomarker
BTC0013	*MLH1*	Deletion	immune checkpoint inhibition	Unknown variant effect

BTC0017	*RAD51B*	Deletion	DDR repair	Unknown variant effect
BTC0016	*MTOR*	Indel	mTOR inhibitor	Unknown variant effect
BTC0027	*IDH2*	Indel	IDH inhibitor	Unknown variant effect
BTC0028	*ERBB2*	Weak amplification	HER2 inhibitor	Unknown variant effect
BTC0035	*MDM2*	Overexpression	MDM2-inhibitor	Unknown variant effect
BTC0043	*FGFR3*	SNV	FGFR inhibitor	Unknown variant effect
BTC0053	*RET*	Overexpression	RET inhibitor	Unknown variant effect
BTC0057	*MTOR*	CNV	MTOR inhibitor	Unknown variant effect
BTC0061	*NF1*	SNV	MEK inhibitor	Unknown variant effect
BTC0064	*FGFR2*	SNV	FGFR inhibitor	Unknown variant effect
BTC0067	*NF1*	Complex knock-out	MEK inhibitor	Unknown variant effect

Figure S1

Legresley Biliary Registry Genomics Research Report for an example case (BTC0010).

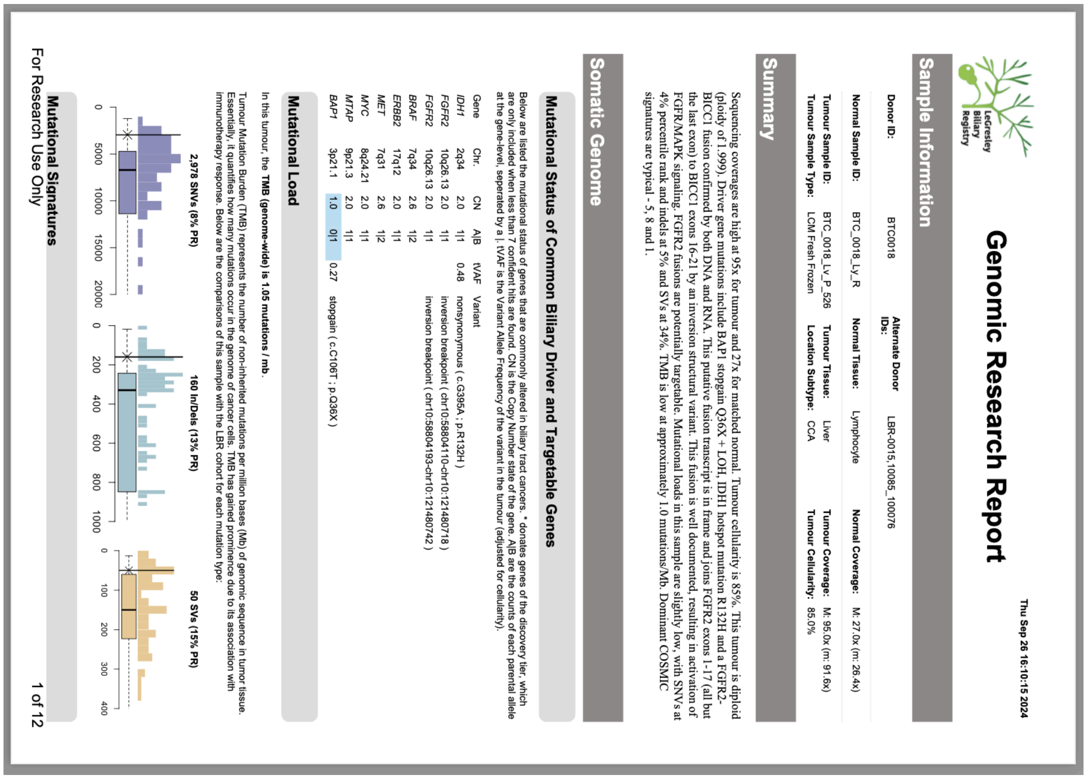

LeGresley Biliary Registry

Thu Sep 26 16:10:15 2024

Genomic Research Report

Sample Information

**Donor ID:**	BTC0018	**Alternate Donor IDs:**	LBR-0015,10085_100076		
**Normal Sample ID:**	BTC_0018_Ly_R	**Normal Tissue:**	Lymphocyte	**Normal Coverage:**	M: 27.0x (m: 26.4x)
**Tumour Sample ID:**	BTC_0018_Lv_P_526	**Tumour Tissue:**	Liver	**Tumour Coverage:**	M: 95.0x (m: 91.6x)
**Tumour Sample Type:**	LCM Fresh Frozen	**Location Subtype:**	CCA	**Tumour Cellularity:**	85.0%

Summary

Sequencing coverages are high at 95x for tumour and 27x for matched normal. Tumour cellularity is 85%. This tumour is diploid (ploidy of 1.999). Driver gene mutations include BAP1 stopgain Q36X + LOH, IDH1 hotspot mutation R132H and a FGFR2-BICC1 fusion confirmed by both DNA and RNA. This putative fusion transcript is in frame and joins FGFR2 exons 1-17 (all but the last exon) to BICC1 exons 16-21 by an inversion structural variant. This fusion is well documented, resulting in activation of FGFR/MAPK signaling. FGFR2 fusions are potentially targetable. Mutational loads in this sample are slightly low, with SNVs at 4% percentile rank and indels at 5% and SVs at 34%. TMB is low at approximately 1.0 mutations/Mb. Dominant COSMIC signatures are typical - 5, 8 and 1.

Somatic Genome

Mutational Status of Common Biliary Driver and Targetable Genes

Below are listed the mutational status of genes that are commonly altered in biliary tract cancers. * donates genes of the discovery tier, which are only included when less than 7 confident hits are found. CN is the Copy Number state of the gene. A|B are the counts of each parental allele at the gene-level, seperated by a |. tVAF is the Variant Allele Frequency of the variant in the tumour (adjusted for cellularity).

Gene	Chr.	CN	A|B	tVAF	Variant
IDH1	2q34	2.0	1|1	0.48	nonsynonymous (c.G395A ; p.R132H)
FGFR2	10q26.13	2.0	1|1		inversion breakpoint (chr10:58804110-chr10:121480718)
FGFR2	10q26.13	2.0	1|1		inversion breakpoint (chr10:58804193-chr10:121480742)
BRAF	7q34	2.6	1|2		
ERBB2	17q12	2.0	1|1		
MET	7q31	2.6	1|2		
MYC	8q24.21	2.0	1|1		
MTAP	9p21.3	2.0	1|1		
BAP1	3p21.1	1.0	0|1	0.27	stopgain (c.C106T ; p.Q36X)

Mutational Load

In this tumour, the **TMB (genome-wide) is 1.05 mutations / mb**.

Tumour Mutation Burden (TMB) represents the number of non-inherited mutations per million bases (Mb) of genomic sequence in tumor tissue. Essentially, it quantifies how many mutations occur in the genome of cancer cells. TMB has gained prominence due to its association with immunotherapy response. Below are the comparisons of this sample with the LBR cohort for each mutation type:

Mutational Signatures

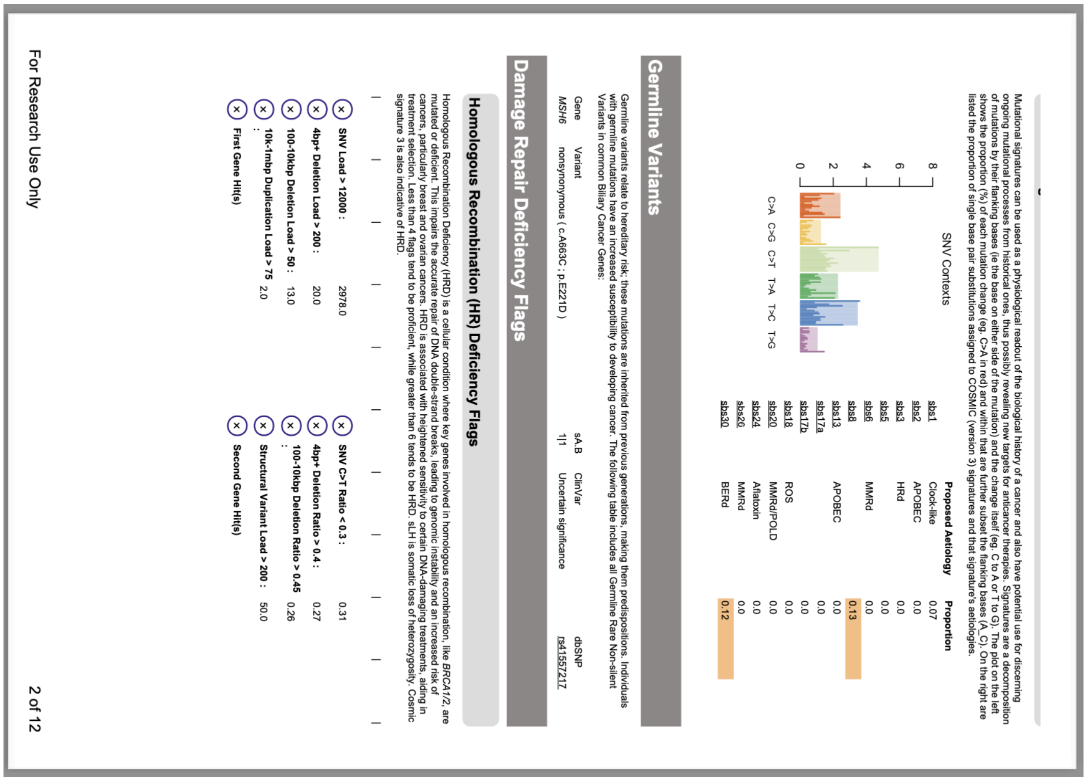

Mutational signatures can be used as a physiological readout of the biological history of a cancer and also have potential use for discerning ongoing mutational processes from historical ones, thus possibly revealing new targets for anticancer therapies. Signatures are a decomposition of mutations by their flanking bases (ie the base on either side of the mutation) and the change itself (eg. C to A or T to G). The plot on the left shows the proportion (%) of each mutation change (eg. C>A in red) and within that are further subset the flanking bases (A_C). On the right are listed the proportion of single base pair substitutions assigned to COSMIC (version 3) signatures and that signature's aetiologies.

	Proposed Aetiology	Proportion
sbs1	Clock-like	0.07
sbs2	APOBEC	0.0
sbs3	HRd	0.0
sbs5		0.0
sbs6	MMRd	0.0
sbs8		0.13
sbs13	APOBEC	0.0
sbs17a		0.0
sbs17b		0.0
sbs18	ROS	0.0
sbs20	MMRd/POLD	0.0
sbs24	Aflatoxin	0.0
sbs26	MMRd	0.0
sbs30	BERd	0.12

Germline Variants

Germline variants relate to hereditary risk; these mutations are inherited from previous generations, making them predispositions. Individuals with germline mutations have an increased susceptibility to developing cancer. The following table includes all Germline Rare Non-silent Variants in common Biliary Cancer Genes:

Gene	Variant	sA.B	ClinVar	dbSNP
MSH6	nonsynonymous (c.A663C ; p.E221D)	1|1	Uncertain significance	rs41557217

Damage Repair Deficiency Flags

Homologous Recombination (HR) Deficiency Flags

Homologous Recombination Deficiency (HRD) is a cellular condition where key genes involved in homologous recombination, like *BRCA1/2*, are mutated or deficient. This impairs the accurate repair of DNA double-strand breaks, leading to genomic instability and an increased risk of cancers, particularly breast and ovarian cancers. HRD is associated with heightened sensitivity to certain DNA-damaging treatments, aiding in treatment selection. Less than 4 flags tend to be proficient, while greater than 6 tends to be HRD. sLH is somatic loss of heterozygosity. Cosmic signature 3 is also indicative of HRD.

⊗ **SNV Load > 12000 :**	2978.0	⊗ **SNV C>T Ratio < 0.3 :**	0.31
⊗ **4bp+ Deletion Load > 200 :**	20.0	⊗ **4bp+ Deletion Ratio > 0.4 :**	0.27
⊗ **100-10kbp Deletion Load > 50 :**	13.0	⊗ **100-10kbp Deletion Ratio > 0.45 :**	0.26
⊗ **10k-1mbp Duplication Load > 75 :**	2.0	⊗ **Structural Variant Load > 200 :**	50.0
⊗ **First Gene Hit(s)**		⊗ **Second Gene Hit(s)**	

Mismatch Repair (MMR) Deficiency Flags

Mismatch Repair Deficiency (MMRd) is a cellular condition marked by impaired DNA mismatch repair mechanisms. This deficiency prevents the correction of errors that occur during DNA replication, leading to the accumulation of mutations and microsatellite instability. MMRd is often associated with Lynch syndrome, a hereditary cancer syndrome, and is associated with microsatellite instability (MSI) or polymerase deficiencies (*POLD/E*). Cosmic signature 20 and 26 are also indicative of MMRD.

✕ **SNV Load > 35000 :**	2978.0	✕ **Indel Load > 3000 :**	160.0
✕ **First Gene Hit(s)**		✕ **Second Gene Hit(s)**	

Tandem Duplicator Phenotype (TDP) Flags

Some (non-BRCA1) tumours are associated with a tandem duplicator phenotypes (TDP) that may be associated with perturbations in *CDK12*, *FBXW7*, and *CCNE1* and predicts platinum and PARP inhibitor sensitivity. Both flags - including a positive Menghi score from Menghi et al. 2018 - are required for a positive TDP call.

✕ **TDP score > 0 :**	-0.51	✓ **Dup. ratio > 0.205 :**	0.24

Gene Expression (WTS)

Transcriptome Quality Control

The RNA from this sample is of **good** quality. We observe:

- **39,341,954** uniquely mapping reads (>3,000,000)
- **38.4 %** unmapped reads (<50%)

Expression Status of Common Biliary Driver and Targetable Genes

Gene	Chr.	CN	TPM	Exp. %ile	Variant
FGFR2	10q26.13	2.0	1779.6	98.7	BICC1::FGFR2 (sense inversion)
PTCH1	9q22.32	2.0	71.0	97.4	upregulated

Additional Information

Figure S2

CONSORT diagram.

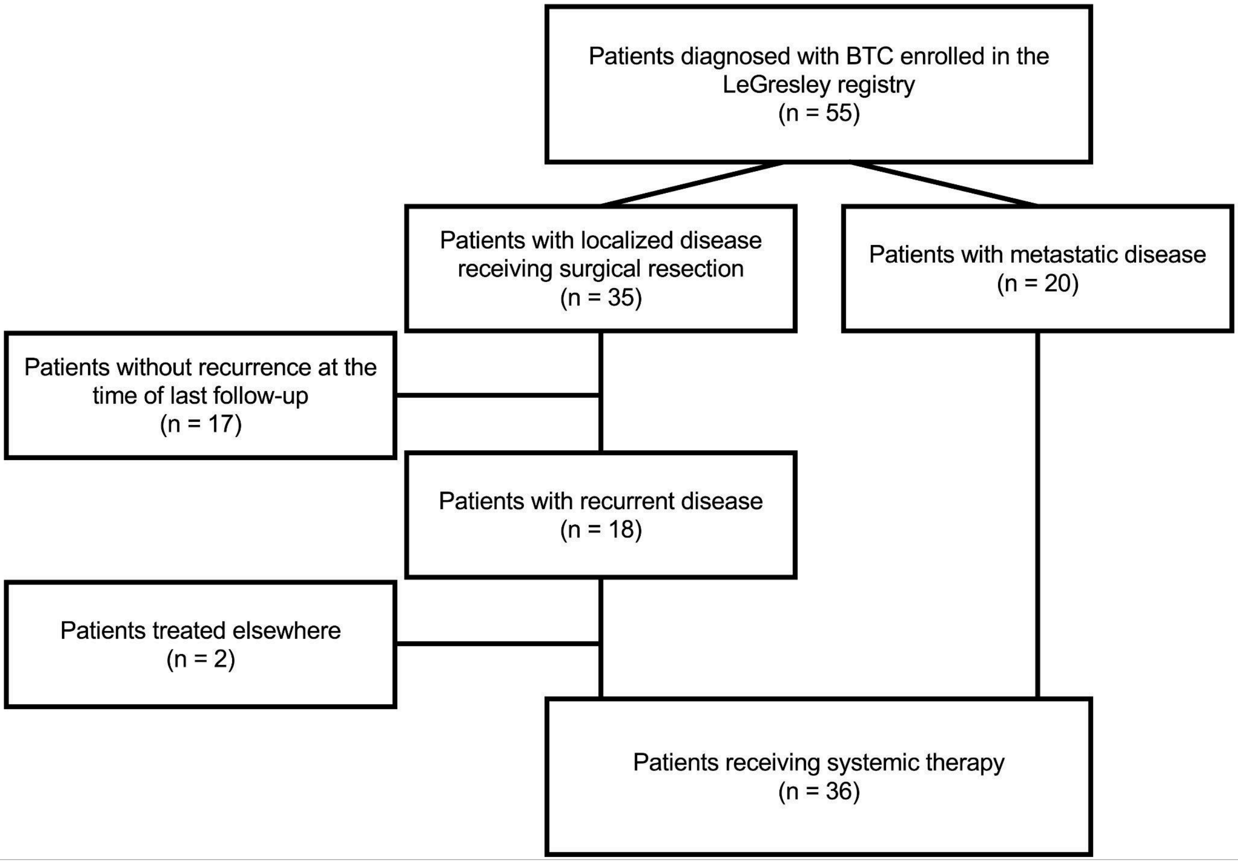

Figure S3

Molecular alterations recommended by the molecular tumor board.

a) Counts and b) identities of molecular alterations recommended by the molecular tumor board compared to OncoKB. MTB: molecular tumor board. TMB: High tumor mutational burden. CNV: copy number variant. HRD: homologous recombination deficiency. SNV: single-nucleotide variant. INDEL: small insertion or deletion. SV: structural variant.

a)

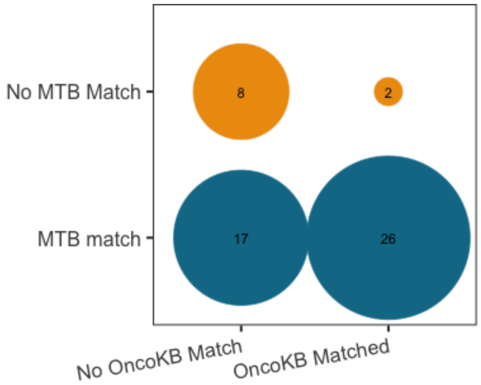

b)

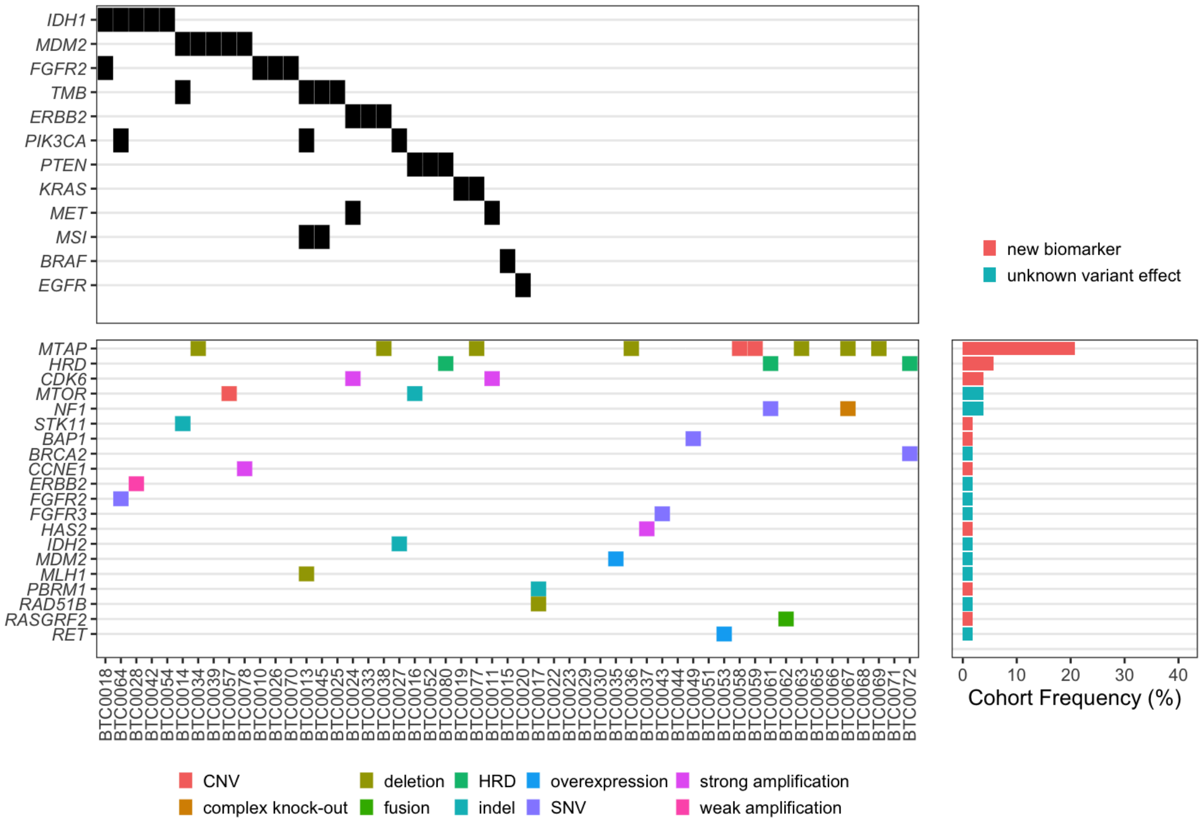

Supplement: Supplementary file 1 [file curroncol-32-00080-s001.zip › curroncol-3417232-supplementary.pdf]
